# Methamphetamine abuse affects gene expression in brain-derived microglia of SIV-infected macaques to enhance inflammation and promote virus targets

**DOI:** 10.1186/s12865-016-0145-0

**Published:** 2016-04-23

**Authors:** Julia A. Najera, Eduardo A. Bustamante, Nikki Bortell, Brenda Morsey, Howard S. Fox, Timothy Ravasi, Maria Cecilia Garibaldi Marcondes

**Affiliations:** Cellular and Molecular Neurosciences Department, The Scripps Research Institute, 10550 North Torrey Pines Rd. SR307, La Jolla, CA 92037 USA; Department of Pharmacology and Experimental Neuroscience, University of Nebraska Medical Center, Omaha, NE 68198-5800 USA; Division of Chemical & Life Sciences and Engineering, King Abdullah University of Science and Technology, Thuwal, 23955-6900 Kingdom of Saudi Arabia; Present address: Northwestern University Feinberg School of Medicine, Chicago, IL 60611 USA

**Keywords:** Methamphetamine, NeuroAIDS, Microglia, CCR5, Simian immunodeficiency virus, Human immunodeficiency virus, Brain, Central nervous system, Inflammation

## Abstract

**Background:**

Methamphetamine (Meth) abuse is a major health problem linked to the aggravation of HIV- associated complications, especially within the Central Nervous System (CNS). Within the CNS, Meth has the ability to modify the activity/function of innate immune cells and increase brain viral loads. Here, we examined changes in the gene expression profile of neuron-free microglial cell preparations isolated from the brain of macaques infected with the Simian Immunodeficiency Virus (SIV), a model of neuroAIDS, and exposed to Meth. We aimed to identify molecular patterns triggered by Meth that could explain the detection of higher brain viral loads and the development of a pro-inflammatory CNS environment in the brain of infected drug abusers.

**Results:**

We found that Meth alone has a strong effect on the transcription of genes associated with immune pathways, particularly inflammation and chemotaxis. Systems analysis led to a strong correlation between Meth exposure and enhancement of molecules associated with chemokines and chemokine receptors, especially CXCR4 and CCR5, which function as co-receptors for viral entry. The increase in CCR5 expression was confirmed in the brain in correlation with increased brain viral load.

**Conclusions:**

Meth enhances the availability of CCR5-expressing cells for SIV in the brain, in correlation with increased viral load. This suggests that Meth is an important factor in the susceptibility to the infection and to the aggravated CNS inflammatory pathology associated with SIV in macaques and HIV in humans.

**Electronic supplementary material:**

The online version of this article (doi:10.1186/s12865-016-0145-0) contains supplementary material, which is available to authorized users.

## Background

NeuroAIDS refers to a series of neurological complications that arise as a result of HIV infection. The effects of NeuroAIDS cannot be prevented by antiviral drugs [1] and, importantly, are aggravated by drugs of abuse such as Methamphetamine (Meth) [2]. Meth is a highly addictive drug, inducing behaviors that increase the users’ risk to HIV exposure [3–5]. HIV-positive individuals that make use of Meth, have a higher incidence of systemic and Central Nervous System (CNS) inflammatory pathology when compared to non-Meth users [2, 6].

Within the CNS, microglia and macrophages are the cells dedicated to immune defense of the brain. These cells regulate CNS physiology and inflammation by secreting cytokines/growth factors and become reactive to CNS/systemic insult [7, 8]. In the post-antiretroviral (ART) era, HIV is mainly a chronic infection, in which microglia exhibit long-lasting changes in gene expression. Such changes may not be simply part of an adaptive microglial response to the virus, but may also result in adverse consequences for neurons and/or synapses. Microglia, which correspond to 10–20 % of the total brain cells [9, 10], are major targets of SIV/HIV virus due to their expression of CCR5 [11]. Identifying changes in brain-isolated and enriched microglial preparations allows for the detection of minor signals that potentially influence immune function in the brain. From these signals we can extrapolate the contribution of the innate immune system in CNS degeneration within the context of HIV infection and aggravation by drug abuse.

Several studies have reported that Meth triggers activation of microglial cells in animal models [12–17]. It has been also reported that Meth users have signs of microglial activation, as seen by the accumulation of the microglial activation marker (1-(2-chrorophynyl)-*N*-methylpropyl)-3 isoquinoline carboxamide ([^11^C]PK11195) [18]. Here, using SIV infected rhesus macaques as a non-human primate model of neuroAIDS, we found that Meth modulates the phenotype of brain innate immune cells by increasing microglial activation and pro-inflammatory cytokine secretion. In addition, Meth also increased CCR5 expression at the surface of innate immune [19]. As a consequence, viral load was also elevated in the brain of Meth-treated animals compared to placebo-treated animals. We further examined the actions of in vivo administration of Meth on the phenotype of microglial cells isolated from the brain of control and SIV-infected macaques. For that, we examined if Meth altered the microglial gene expression profile in a way that could elucidate the enhanced severity of virus-triggered tissue-pathology that is observed in HIV+ Meth abusers. Our results demonstrate that Meth alters the pro-inflammatory phenotype of brain innate immune cells and this effect is enhanced in the context of SIV infection. Thus, our data suggests that Meth alters the microglial gene expression profile in a way that can explain the enhanced severity of virus-triggered tissue pathology in HIV positive Meth users.

## Results and discussion

### A. High score pathways and most up-regulated genes in brain-isolated immune cells

**Fig. 1:**
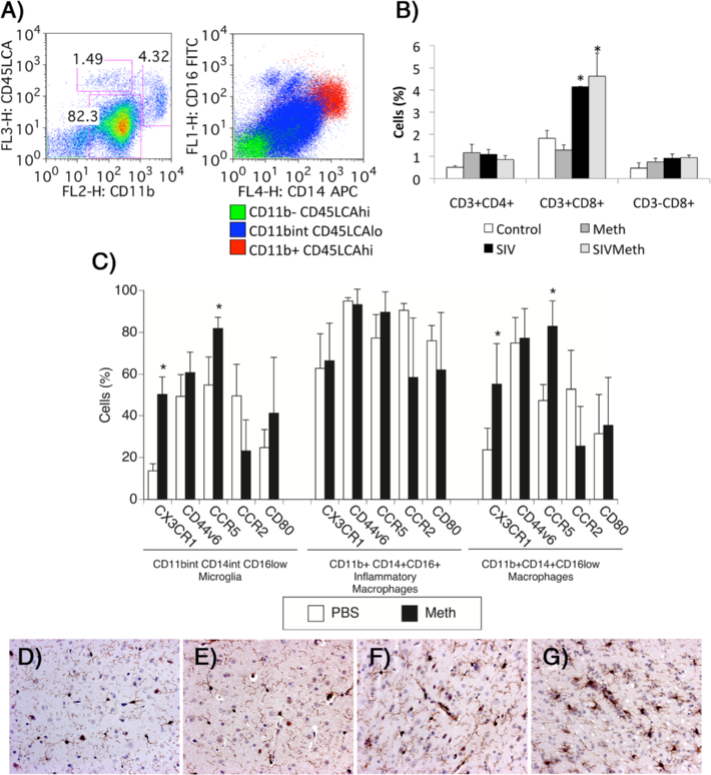
Characterization of cell subpopulations in the brain-derived cell isolates. Percoll-isolated cells from the brain of rhesus macaques were utilized in this study. These cells were characterized based on the expression of surface markers of cell subpopulation, and analyzed flow cytometry. **a** Cell gating strategies. The cells were gated based on the expression of CD11b (myeloid cell marker) and CD45LCA (peripheral cell marker), where CD11bint CD45LCA- cells are microglia, CD11b + CD45LCA+ cells are macrophages and CD11b-CD45LCA+ cells are mostly lymphocytes. The identity of microglia and macrophages was further confirmed by the CD14 and CD16 expression patterns. **b** Percentage of Lymphoid cells. The CD11b- population was characterized based on the expression of lymphocyte markers CD3, CD4 and CD8, and the relative differences in the percentage of lymphocyte subsets between groups is shown. *p ≤ 0.05 compared to uninfected controls. **c** Myeloid cell subset characterization. Myeloid cell subsets were differentially analyzed based on the expression of inflammatory status surface markers, CX3CR1, CD44v6, CCR5, CCR2 and CD80. *p ≤ 0.05 compared to uninfected controls. Microglia activation: Immunohistochemical detection of Iba-1 microglial marker in brains from animals that were (**d**) Controls, (**e**) SIV-infected, (**f**) Meth-treated, (**g**) SIV-infected and Meth-treated. Sections from the frontal cortex were examined for the frequency and distribution of Iba-1-expressing cells. The panels show one representative animal per group

We have profiled changes in gene expression of brain-derived CD11b-enriched immune cells (Fig. 1a), in healthy (control *n* = 5) and SIV-chronically infected macaques, treated (SIV/Meth *n* = 4) or not with Meth (SIV only *n* = 4). Uninfected animals that received Meth were also included as abuse controls (Meth only *n* = 4). For comparison, we also profiled genes from microglia isolated from animals exhibiting encephalitis associated to disease progression (SIVE *n* = 4). All infected animals had equivalent and stable SIV viremia [19], when Meth treatment was initiated post acutely. This approach allowed the identification of changes in the microglial phenotype that were driven by the introduction of drug. Importantly, and as described previously [19], the introduction of Meth did not affect viral load in the plasma in comparison to the SIV group.

The characterization of the brain-derived innate immune cells analyzed in this study was performed using flow cytometry and cell subset surface markers, such as CD45LCA (which characterizes cells that have a peripheral origin), CD11b (which characterizes innate immune cells) (Fig. 1a left), in addition to other innate immune cell markers CD14 and CD16, which estimate the inflammatory status when plotted together (Fig. 1a right). In Fig. 1a, a representative sample exemplifies the gating strategy used in this study, based on the distribution of CD45LCA and CD11b-positive cells (Fig. 1a left), and showing the general aspect of brain preparations in flow cytometry. The majority of the cells in the isolates were CD11bint/+ CD45LCAlow cells microglia, followed by a subpopulation of CD11b + CD45LCAhi macrophages and a CD11bint CD45LCAhi subset (containing <2 % CD19+ B cells- and <4 % CD11c + myeloid dendritic cells - not shown). On the other hand, within the gated CD11bint CD45lo microglia cells, the majority expressed CD16lo and CD14int levels, but a small population expressing CD16hi was also identified. The CD11b + CD45LCAhi macrophages were CD14 hi, but these cells had a range of CD16 expression levels, from intermediate to high. For the most part, this cell distribution pattern was maintained throughout the groups.

Within the CD45LCA+ cells, we have also examined the presence of CD11b-negative cells, expressing CD3 (a marker of T lymphocytes), together with either CD4 (a marker of T helper cells) or CD8 (a marker of NK and cytotoxic T cells) (Fig. 1b). Among these lymphoid subsets, SIV infection induced an enrichment of cytotoxic T cells (Fig. 1b), as expected. In addition, changes in relative numbers of CD3 + CD8+ T cells, but not other lymphoid cells, were due to SIV infection and were not affected by Meth (Fig. 1b). However, we have previously shown that Meth does increase the activation of CD3 + CD8+ cells and CD3-CD8+ NK cells [19].

The CD11b-positive cells expressing CD14 and different levels of CD16, were further analyzed according to inflammatory markers that are relevant in the SIV/HIV neuropathogenesis, such as the Osteopontin receptor CD44v6, chemokine receptors CX3CR1, CCR5 and CCR2, and the activation marker CD80 (Fig. 1c). Although the overall percentages of the most dominating innate immune cell subsets, such as microglia and macrophages, were not significantly affected by Meth, we did observe changes in the expression of activation markers (Fig. 1c). For example, we detected an increase in levels of chemokine receptors CX3CR1 and CCR5 on macrophages (Fig. 1c). Interestingly, this effect was detected, particularly, on cells displaying characteristics of resident microglia, as well as on the macrophage subset that was CD14-positive but still retained the CD16low phenotype (Fig. 1c), suggesting the onset of cells with an intermediate inflammatory phenotype. The activation of microglia was largely detectable by histopathology and immunohistochemistry, using the Iba-1 antibody, which detects the Allograft Inflammatory Factor 1 (AIF1) – a marker of microglial activation (Fig. 1d-g). For instance, the histopathological findings suggested that in the presence of SIV microglial cells, which are the majority of the cell isolate, increased the expression of Iba-1 (Fig. 1e and f, respectively) compared to controls (Fig. 1d). In addition, the combination of Meth and SIV infection potentiated the increase of Iba-1 expression (Fig. 1g), suggesting that high microglial activation induced by SIV is enhanced by Meth. The increase in Iba-1 expression was confirmed by image analysis using ImageJ, where thresholded stained particles further adjusted to eliminate counterstaining signal, were converted into brightness and quantified. Using this strategy Iba-1 occurred in 5.58 % of the control frontal cortex tissue (±0.06), with a significant increase to 7.16 % (±0.18, *p* = 0.021, using Bonferroni’s post hoc test) due to SIV infection. Meth-treated animals presented 6.43 % (±0.010) of the frontal cortex area stained with Iba-1 antibody, which was significant compared to controls (*p* = 0.029). In SIV/Meth animals we found that 8.78 % of the frontal cortex area was Iba-1-positive (*p* = 0.045 compared to SIV alone). The increase in Iba-1 staining in SIV/Meth was accompanied by an increase in the frequency of perivascular inflammatory cells as well as an increase in the size of cells with microglia morphology, thereby shifting the inflammation from mild to moderate. Thus, all infected animals exhibited signs of inflammatory response, which was characterized as mild in SIV (increased Iba-1 expression, and presence of perivascular as well as diffuse macrophages), and moderate in the presence of SIV plus Meth (stronger Iba-1 expression, intensified perivascular and submeningeal infiltrate, presence of parenchymal macrophage foci, and occasional giant cells).

Following the characterization of these brain-derived CD11b-rich immune cells, the isolates were processed for gene array and differences in gene expression between groups were mapped to establish the fingerprints of SIV and Meth abuse within the innate immune and lymphoid populations. Following hybridization procedures, the differences between groups were detectable upon visual inspection of hybridized microarrays. Absolute signal intensity ratios were estimated between paired groups. The complete expression dataset was then mapped, and groups were compared. Hotelling confidence interval calculation did not identify outliners, and comparisons were performed using both ANOVA and a two-factor general linear model.

**Fig. 2:**
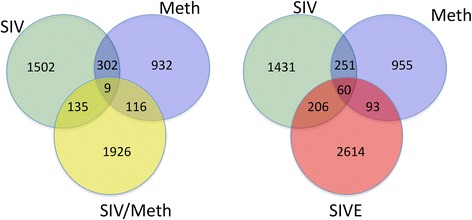
Venn diagram indicating the number of significantly upregulated genes in SIV, Meth and SIV/Meth groups, as well as SIV, Meth and SIVE animals. Genes represented were increased above 1.5 fold with a *p* value ≤ 0.05 in comparisons

The significant changes in gene expression due to SIV and/or Meth were analyzed for identification of gene signatures, which were further analyzed using an integrative systems biology approach described as follows. By filtering the data to consider only genes that were up-regulated to above 1.5-fold in group comparisons, with a *p* value <0.05, the number of genes that were changed in different conditions was as follows: Meth treatment alone significantly up-regulated 1359 genes compared to Controls; SIV infection increased 1948 genes in isolated microglia compared to controls. The introduction of Meth treatment in SIV-infected macaques induced the up-regulation of 481 genes in comparison to SIV alone, and of 715 genes in comparison to Meth alone. In addition, there were 311 genes up-regulated in both Meth alone and in SIV alone, of which 9 were also upregulated in SIV/Meth, and 60 have been also found in microglia from animals exhibiting disease progression and encephalitis encephalitis. A visual representation of the number of upregulated genes in individual groups can be found in Fig. 2.

Pathway assignments and functional annotations were analyzed using DAVID Bioinformatics Database [20], As well as Ingenuity Knowledge Base [21] and an interaction repository, which is based on cpath [22–24] and includes interactions that have been curated by GeneGo (http://portal.genego.com) and Ingenuity. Networks retrieved from the latter were visualized using Cytoscape [25]. Both resources were queried using Markov clustering (MCL) algorithm, to infer how the derived differential expression data may interact with established Gc pathways. This approach was utilized to facilitate the visualization of Meth’s interference on molecular patterns triggered by the virus. We examined a select number of pathways based on their score and relevance to immune pathology.

The genes up-regulated by each condition in comparison to controls were clustered for functional annotation using DAVID Bioinformatics Database and the 15 most upregulated genes in each group were highlighted (Tables 1, 2, 3, 4, and 5). In Cytoscape, pathways were scored following the application of Markov clustering (MCL) algorithms, and nodes were obtained according to the number of assigned up-regulated genes using Cytoscape interface. Pathways with four or more up-regulated genes are reported. Meth significantly affected genes of the immune system and metabolic signaling pathways, suggesting the drug deeply modifies microglia cells.

**Table 1:** Functional annotation chart for microglia gene pathways that were significantly up-regulated by Meth in microglia, as compared to controls. Number of genes, *p*-value and Benjamini value are presented. Complete data set is presented in Additional file 1: Table S1

Category	Term	Count	*P*-value	Benjamini
KEGG_PATHWAY	Cytokine-cytokine receptor interaction	24	9.00E-07	1.30E-04
GOTERM_BP_FAT	Immune response	18	1.90E-03	2.50E-01
GOTERM_CC_FAT	Extracellular region part	15	1.60E-02	6.30E-01
GOTERM_MF_FAT	Cytokine activity	14	1.80E-03	1.90E-01
GOTERM_CC_FAT	Extracellular space	14	2.10E-02	4.90E-01
KEGG_PATHWAY	Natural killer cell mediated cytotoxicity	13	8.10E-04	5.50E-02
KEGG_PATHWAY	Chemokine signaling pathway	13	3.20E-02	2.90E-01
GOTERM_BP_FAT	Defense response	12	1.40E-02	4.60E-01
KEGG_PATHWAY	Jak-STAT signaling pathway	12	1.80E-02	2.00E-01
KEGG_PATHWAY	T cell receptor signaling pathway	10	1.50E-02	2.10E-01
KEGG_PATHWAY	Hematopoietic cell lineage	9	1.10E-02	1.80E-01
KEGG_PATHWAY	Toll-like receptor signaling pathway	9	2.30E-02	2.40E-01
KEGG_PATHWAY	NOD-like receptor signaling pathway	8	5.50E-03	1.40E-01
KEGG_PATHWAY	Allograft rejection	8	7.40E-03	1.60E-01
GOTERM_BP_FAT	Response to wounding	8	1.30E-02	5.10E-01
KEGG_PATHWAY	Fc epsilon RI signaling pathway	8	1.70E-02	2.10E-01
KEGG_PATHWAY	Asthma	7	2.00E-03	6.80E-02
GOTERM_BP_FAT	Inflammatory response	7	7.90E-03	3.90E-01
KEGG_PATHWAY	Cytosolic DNA-sensing pathway	7	8.20E-03	1.50E-01
KEGG_PATHWAY	Autoimmune thyroid disease	7	5.70E-02	4.20E-01
GOTERM_BP_FAT	Chemotaxis	6	1.40E-02	4.20E-01
GOTERM_MF_FAT	Cytokine binding	6	3.40E-02	8.60E-01
GOTERM_BP_FAT	Locomotors behavior	6	3.40E-02	7.10E-01
KEGG_PATHWAY	Graft-versus-host disease	6	4.30E-02	3.60E-01
INTERPRO	Immunoglobulin	6	4.50E-02	9.00E-01
KEGG_PATHWAY	Type I diabetes mellitus	6	6.90E-02	4.70E-01
GOTERM_BP_FAT	Behavior	6	7.90E-02	9.20E-01
GOTERM_BP_FAT	Cell migration	5	1.80E-03	4.30E-01
GOTERM_BP_FAT	Cell motility	5	3.30E-03	2.90E-01
INTERPRO	Small chemokine, C-C group, conserved site	5	5.50E-02	8.80E-01
GOTERM_BP_FAT	Leukocyte migration	4	6.40E-03	4.00E-01
INTERPRO	Chemokine receptor	4	6.40E-02	8.70E-01
GOTERM_MF_FAT	C-C chemokine receptor activity	4	7.20E-02	9.40E-01
GOTERM_MF_FAT	C-C chemokine binding	4	7.20E-02	9.40E-01
GOTERM_MF_FAT	Chemokine receptor activity	4	9.50E-02	9.40E-01

**Table 2:** Fifteen most up regulated genes in Meth-treated macaques in comparison to controls

Gene	Name	Fold change	*P* value
GQ153436	MHC Class I Mamu-B	5.43	0.0001
MAMU-DQ	MHC Class II DQ variant	4.76	0.01
FOLR1	Folate receptor 1	4.76	0.044
CCL5	Chemokine C-C motif ligand 5	3.66	0.0021
GPR160	G-protein coupled receptor 160	3.32	0.006
IGFBP1	Insulin-like growth factor binding protein 1	3.21	0.0007
PTGER2	Prostaglandin E receptor 4	2.91	0.01
CXCR2	CXC chemokine receptor 2 (IL8RB)	2.77	0.009
CCL3	Macrophage Chemoatractant Protein-1	2.63	0.03
TGFBI	Transforming growth factor beta-induced	2.6	0.005
IL4	Interleukin 4	2.58	0.016
ALOX5AP	Arachidonate-5-lipoxygenase activating protein	2.51	0.023
IL10	Interleukin 10	2.38	0.015
INFB1	Interferon beta 1	2.35	0.025
CCR5	Chemokine C-C motif receptor 5	2.24	0.0029

**Table 3:** Functional annotation chart for the gene pathways that were significantly up-regulated by chronic SIV in microglia, as compared to controls. Number of genes, *p*-value and Benjamini value are presented. Complete data set is provided in Additional file 2: Table S2

Category	Term	Count	*P*-value	Benjamini
GOTERM_CC_FAT	Intrinsic to membrane	25	1.20E-02	1.60E-01
GOTERM_CC_FAT	Integral to membrane	24	1.60E-02	1.60E-01
KEGG_PATHWAY	Endocytosis	20	7.80E-03	1.10E-01
SP_PIR_KEYWORDS	Transmembrane	20	8.70E-02	8.70E-01
GOTERM_BP_FAT	Immune Response	19	2.10E-04	2.00E-02
KEGG_PATHWAY	Cell adhesion molecules (CAMs)	19	1.00E-03	2.00E-02
KEGG_PATHWAY	Cytokine-cytokine receptor interaction	18	6.30E-02	4.20E-01
INTERPRO	Immunoglobulin-like fold	17	2.10E-06	2.40E-04
KEGG_PATHWAY	Lysosome	17	1.00E-04	5.40E-03
KEGG_PATHWAY	Natural killer cell mediated cytotoxicity	17	5.30E-04	1.20E-02
KEGG_PATHWAY	Antigen processing and presentation	16	1.10E-04	3.50E-03
GOTERM_CC_FAT	Plasma MEMBRANE	16	2.50E-02	1.70E-01
KEGG_PATHWAY	Viral myocarditis	15	1.70E-03	3.00E-02
KEGG_PATHWAY	Allograft rejection	14	3.20E-05	2.60E-03
KEGG_PATHWAY	Autoimmune thyroid disease	14	2.40E-04	6.40E-03
KEGG_PATHWAY	Graft-versus-host disease	13	2.60E-05	4.20E-03
KEGG_PATHWAY	Type I diabetes mellitus	13	1.00E-04	4.20E-03
GOTERM_BP_FAT	Antigen processing and presentation	10	5.20E-05	1.40E-02
GOTERM_CC_FAT	MHC protein complex	10	5.80E-05	2.50E-03
GOTERM_CC_FAT	Plasma Membrane Part	10	2.20E-02	1.80E-01
KEGG_PATHWAY	Colorectal cancer	10	5.80E-02	4.20E-01
KEGG_PATHWAY	Cytosolic DNA-sensing pathway	9	5.30E-03	8.20E-02
KEGG_PATHWAY	Fc gamma R-mediated phagocytosis	9	9.80E-02	5.50E-01
KEGG_PATHWAY	Acute myeloid leukemia	8	2.50E-02	2.70E-01
KEGG_PATHWAY	Amyotrophic lateral sclerosis (ALS)	8	4.80E-02	3.90E-01
KEGG_PATHWAY	Intestinal immune network for IgA production	8	4.80E-02	3.90E-01
GOTERM_CC_FAT	MHC class I protein complex	7	5.80E-04	1.20E-02
SP_PIR_KEYWORDS	Immune response	7	1.40E-03	6.20E-02
INTERPRO	Immunoglobulin subtype	7	1.00E-02	1.40E-01
KEGG_PATHWAY	Amino sugar and nucleotide sugar metabolism	7	2.40E-02	2.80E-01
GOTERM_BP_FAT	Antigen processing and presentation of peptide antigen via mhc class i	6	7.00E-05	9.80E-03
INTERPRO	MHC class I-like antigen recognition	6	7.90E-03	1.20E-01
KEGG_PATHWAY	Primary immunodeficiency	6	3.90E-02	3.70E-01
KEGG_PATHWAY	Sphingolipid metabolism	6	5.50E-02	4.20E-01
GOTERM_BP_FAT	Cell death	5	2.60E-02	8.40E-01
KEGG_PATHWAY	RNA polymerase	5	4.50E-02	3.90E-01
GOTERM_BP_FAT	Apoptosis	4	9.50E-02	9.90E-01

**Table 4:** Fifteen most up regulated genes in isolated microglia from SIV-infected macaques in comparison to controls

Gene	Name	Fold change	*P* value
FCN1	Ficolin (collagen/fibrinogen domain containing) 1	20.26	0.01837
APOBEC3A	Apolipoprotein B mRNA editing enzyme catalytic polypeptide-like 3A	15.34	0.04385
GZMA	Granzyme A	14.96	0.04000
MAMU-A	MHC class I antigen	10.64	0.00076
GZMB	Granzyme B	10.02	0.00454
LPAR5	lysophosphatidic acid receptor 5	9.63	0.04561
NKG7	Natural killer cell group 7 sequence	9.11	0.02109
PDCD1LG2	Programmed cell death 1 ligand 2	9.11	0.02552
GPR18	G protein-coupled receptor 18	8.09	0.01031
LFA2	Lymphocyte function-associated antigen 2	7.74	0.00447
TIGIT	T cell immunoreceptor with Ig and ITIM domains	7.53	0.04879
CHRNA1	Cholinergic receptor, nicotinic, alpha 1	7.28	0.04968
CLEC2D	C-type lectin domain family 2, member D	7.01	0.03784
GLIPR1	GLI pathogenesis-related 1	6.54	0.03590
BTLA	B- and T-lymphocyte attenuator-like	6.32	0.00352

**Table 5:** Fifteen most up regulated genes in SIV/Meth macaques in comparison to SIV

Gene	Name	Fold change	*P* value
AQP9	Aquaporin 9	94.2	0.028
OAS1	2′,5′-oligoadenylate synthetase 1	11.98	0.00005
MAMU-A1	Macaca mulatta Mamu-A MHC class I antigen	5.19	0.038
GPD1	Glycerol-3-phosphate dehydrogenase 1	3.69	0.037
SERPINA1	Serpin peptidase inhibitor, clade A	3.37	0.002
VSIG1	V-set and immunoglobulin domain containing 1	3.1	0.02
NAT2	N-acetyltransferase 2 (arylamine N-acetyltransferase)	2.96	0.026
CD244	Natural killer cell receptor 2B4	2.78	0.031
IRF5	Interferon regulatory factor 5	2.66	0.032
TXNDC2	Thioredoxin domain-containing protein 2-like	2.59	0.028
IL1RAPL1	Interleukin 1 receptor accessory protein-like 1	2.29	0.007
GRIN2B	Glutamate receptor, ionotropic, N-methyl D-aspartate 2B	2.29	0.003
ZSCAN1	Zinc finger and SCAN domain containing 1	2.17	0.023
GPR17	G protein-coupled receptor 17, transcript variant 2	2.13	0.016
HIVEP3	Human immunodeficiency virus type I enhancer binding protein 3	94.21	0.028

### A1. Meth alone upregulates pathways and genes involved in signaling and in immune functions

Meth significantly increased immune system and metabolic signaling pathways (Table 1). Table 2 shows the most up-regulated genes in Meth-treated animals in comparison to uninfected controls. These genes have been assigned to pathways involved in a broad number of pathways, such as in disulfide bonds, cell surface receptor-linked signal transduction, chemotaxis, and responses to wounding, regulation of cell proliferation, immune and inflammatory responses.

### A2. SIV alone upregulates pathways and genes associated with the CNS viral response

The genes up-regulated in isolated microglial cells by SIV alone in comparison to controls were clustered for functional annotation using DAVID Bioinformatics Database (Table 3), and pathways were scored according to the number of assigned up-regulated genes. Pathways with a count of four or more up-regulated genes are reported. As expected, the microglial genes that were upregulated by SIV in comparison to uninfected controls were related to the viral response in the CNS, including antigen processing/presentation and cell death (Table 3).

Table 4 shows the fifteen most up-regulated genes in SIV-infected brain-derived microglia compared to control, which have been assigned to pathways associated to plasma membrane (*p* = 0.0005, Benjamini = 0.001), immune response (*p* = 0.0008, Benjamini = 0.008), negative regulation of the immune process (*p* = 0.0003, Benjamini = 0.003), apoptosis (*p* = 0.0002, Benjamini = 0.004), and regulation of leukocyte proliferation (*p* = 0.005, Benjamini = 0.03).

### A3. SIV/Meth upregulates pathways and genes involved in metabolism and immune function

We next examined the genes that were significantly upregulated by Meth in SIV-infected brain-derived microglia (SIV/Meth) in comparison to SIV alone, as described above. In SIV-infected macaques, the introduction of Meth significantly affected genes involved in neuroactive synapsis interaction (*p* = 0.002, Benjamini = 0.008).

The fifteen most up-regulated genes in SIV/Meth microglia, compared to SIV, showed assignments to specific pathways involved in to immunoglobulin domain (*p* = 0.003, Benjamini = 0.08), Toll-like receptor response (*p* = 0.004, Benjamini = 0.01), alternative splicing (*p* = 0.007, Benjamini = 0.07), glycoproteins (*p* = 0.008, Benjamini = 0.07), and receptor signaling (*p* = 0.009, Benjamini = 0.06) (Table 5).

### B. Microglial gene network visualization

**Fig. 3:**
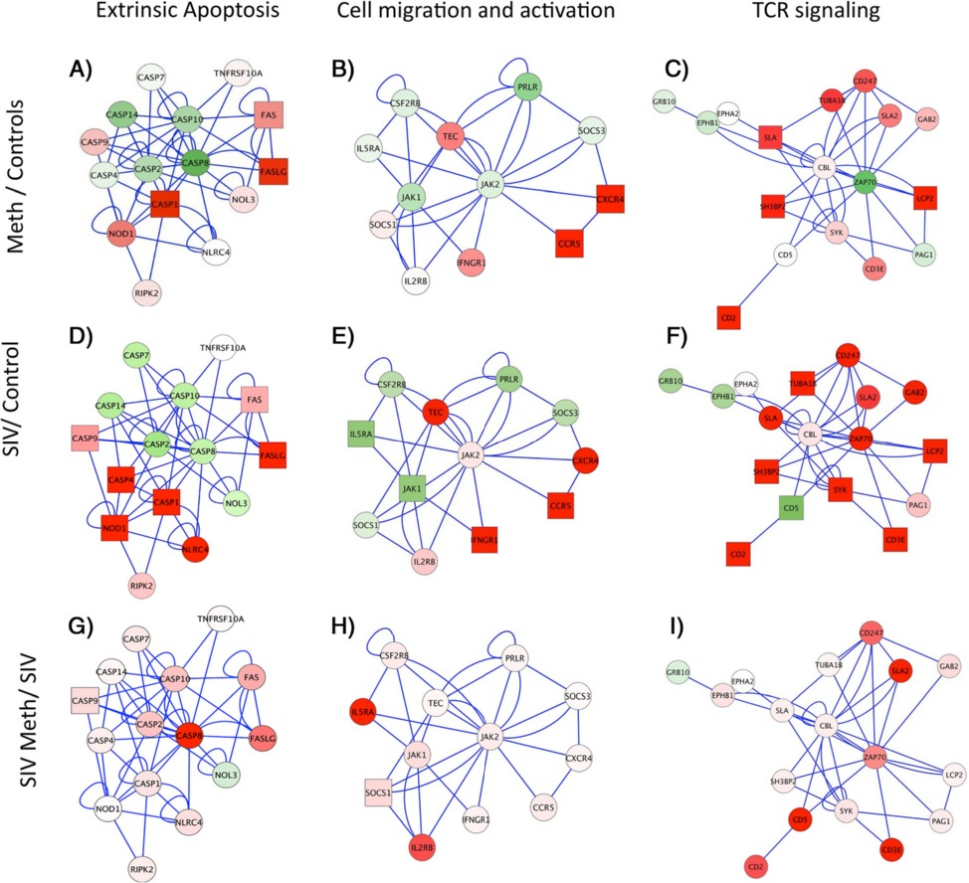
Highest scoring significant modules associated to immune functions in microglia from Meth-treated macaques. Comparisons between Meth and controls (**a, b, c**), SIV and controls (**d, e, f**), and SIV/Meth and SIV (**g, h, i**) were performed using Cytoscape interface, and Biogrid plugin. Data was filtered based on fold-change above 1.5-fold and *p* value <0.05. Clustered gene nodes were selected to present at least two upregulated genes in Meth compared to controls, and to have a described role affecting CNS immune functions and viral load. These filtered nodes were related to extrinsic apoptotic pathways (**a, d, g**), T-cell receptor (TCR) signaling (**b, e, h**), and macrophage and microglia activation and chemotaxis (**c, f, i**). Red color intensity signifies upregulation levels, and green signifies down-regulation. Shapes indicate significance, being squares *p* < 0.05 and circles >0.05

Gene networks were visualized using Cytoscape [25], and queried using Markov Clustering (MCL) algorithms, to infer how the derived differential expression data may interact with established pathway databases. This approach was utilized to facilitate the visualization of the influence of Meth on molecular patterns triggered by the virus. We focused on comparisons between Meth and Control groups (Fig. 3a, b and c), to select high score nodes, filtered for a minimum of 3 genes showing similar behavior, such as expression above 1.5-fold increase, and a *p* value ≤0.05. We analyzed these changes in parallel with changes observed in SIV only compared to controls (Fig. 3d, e, and f) and finally selected nodes where the combination of Meth and SIV showed enhanced expression of genes compared to SIV alone (Fig. 3g, h and i) and that could have implications in inflammatory outcome, enhancement of brain viral load, and progression. This analysis led to three networks with a role in cell survival and immune functions, which were extrinsic apoptosis (Fig. 3a, d and g), cell migration/activation (Fig. 3b, e and h), and T-cell receptor (TCR) signaling (Fig. 3c, f and i).

Regarding the extrinsic apoptosis pathway, we found that Meth significantly upregulated genes such as Fas (CD95, 1.53 fold, *p* = 0.0051),Fas LG (1.95 fold, *p* = 0.044), and Caspase 1 (2.07 fold, *p* = 0.022) compared to controls (Fig. 3a). Also in this pathway, NOD1 (1.6-fold, *p* = 0.09) and Caspase 9 (1.58-fold, *p* = 0.062) were substantially but not significantly increased. Genes in this pathway, such as Fas, Fas LG, NOD1, Caspases 1, 9 and 4 were significantly increased by SIV in comparison to controls (Fig. 3d). Meth and SIV caused a significant 1.5-fold up-regulation (*p* = 0.01) of Caspase 9 when compared to SIV alone (Fig. 3g).

Apoptotic pathways that involve the upregulation of CD95 have been previously implicated in the progression of neuroAIDS [26]. We have identified the upregulation of Fas as a characteristic of active immune cells in the brain of SIV-infected macaques [27]. In microglia, the upregulation of Fas correlates with activation after infection, as a response to local increases in TNFα and IFNγ [28]. Our results indicate that in both Meth-treated and SIV-infected brain isolates, this pathway affects Caspase 1 levels. SIV alone increases transcription of Caspases 4 and 9. However, the combination of SIV and Meth enhances the transcription of Caspase 9 compared to SIV alone, which is within the non-canonical Caspase 8-dependent pathway [29, 30]. While Caspase 1 (along with Caspase 4) is a key mediator of inflammation [31, 32], Caspase 8 and 9 may commit cells to apoptosis [33–35]. Suggesting that in animals infected with SIV and treated with Meth inflammation can be accompanied by loss of glial cells. Indeed, pathological analyses of HIV-1-infected brains have shown that apoptosis is observed both in neurons and in microglia [36, 37] and that chronic Meth enhances this process [26].

We also examined a pathway that is downstream of T cell receptor-mediated activation of T cells, which is centered in the expression of Zap70 tyrosine kinase [38]. This pathway seemed relevant, not only for a high score upon pathway analyses, but also because CD8 T cells can be found in the microglial fraction isolated from brain [27], and are increased by SIV infection (Fig. 1b). Although the numbers of CD4+ T cells and especially of CD8+ T cells are not affected by Meth, changes in this pathway suggest that Meth did affect T cell activation and response (Fig. 3b). For instance, Meth substantially decreased the expression of a central component in TCR signaling, Zap 70 (0.5-fold, *p* = 0.08), in correlation with the significant upregulation of SLA (Src-like adaptor) (1.8-fold, *p* = 0.011), which negatively regulates TCR signaling [39]. LCP2 (Lymphocyte cytosolic protein 2)(2.1-fold, *p* = 0.008), SH3BP2 (SH3-domain binding protein 2)(1.93-fold, *p* = 0.05), and CD2 (LFA2)(12.85-fold, *p* = 0.045) were also significantly upregulated by Meth. As expected, this pathway was highly upregulated by SIV infection in comparison to controls (Fig. 3e). In addition, the combination of Meth and SIV substantially enhanced the expression of components of this node (Fig. 3h).

The ability of both Meth and SIV alone to upregulate the substrate of Zap70 protein, LCP2 (Lymphocyte cytosolic protein 2), the cooperating molecule SH3BP2 (SH3-domain binding protein 2), and the early T cell differentiation marker CD2, could be due to changes in the absolute numbers of CD8 T cells. However, neither SIV nor Meth was able to significantly change the expression of Zap70. Meth alone increased the expression of the Zap70-cooperating molecule SLA (Src-like adaptor), suggesting an effect on the adaptative branch of the immune response. SIV alone triggered the expression of CD3ε, Syk and TUBA1B (Tubulin alpha), suggesting TCR mediated antigen recognition, activation, and cytoskeleton organization [40]. Importantly, the combination of Meth and SIV did not enhance this T cell receptor-mediated pathway compared to SIV alone. Thus confirming that the major additive and synergistic effects of Meth and SIV occur in innate immune cells of the brain.

Meth also upregulated pathways involved in chemotaxis of immune cells as well as microglial motility. For instance, Meth increased the expression of CCR5 by 2.24-fold (*p* = 0.01) and CXCR4 by 2.29-fold (*p* = 0.01) (Fig. 3b). This data is in accordance with previous observations suggesting that Meth increases the availability of virus target cells. These same genes were upregulated by SIV, in addition to IFNGR1, when compared to controls (Fig. 3e). In SIV/Meth microglia, the upregulation of CCR5 and CXCR4 did not differ in comparison to SIV. However, the expression of another gene in this pathway, SOCS1 (Suppressor of cytokine signaling 1), a marker of DNA damage and senescence [41], was significantly increased in SIV/Meth microglia (1.73-fold, *p* = 0.05). Within the same pathway, Meth in SIV-infected brain isolates substantially upregulated the expression of IL2RG (6.7-fold, *p* = 0.26) (Fig. 2i).

### C. Increased expression of inflammatory and viral target molecules in Meth microglia

The identification of cell migration pathways in association with Meth exposure raised a special interest, not only from the inflammatory perspective, but also from the fact that the surface molecules that facilitate the entry of the SIV and HIV viruses into host cells (CXCR4 and CCR5) were found to be highly upregulated by Meth in our gene arrays. This finding is in accordance with our previous study, which suggested that CCR5 upregulation is a marker of disease severity associated with Meth use in the CNS of SIV-infected macaques [19]. The in-depth examination of this pathway could lead to the understanding of mechanisms by which Meth aggravates CNS infection and outcome. Thus, we examined genes associated with Meth-induced CCR5 upregulation in microglia by tracking other molecular components associated with CCR5 upregulation by Meth. Importantly, the combination of SIV and Meth caused the upregulation of SOCS1 (Suppressor of cytokine signaling 1). SOCS1 can directly inhibit the JAK kinase activation loop and signaling through interferon (IFN) receptors [42], which may contribute to the development of an immune suppression state. Interestingly, the expression of SOCS1 is directly correlated to the development of immune senescence [41]. In parallel, CCR1 and RANTES were also drastically elevated in animals where Meth and SIV were combined.

**Fig. 4:**
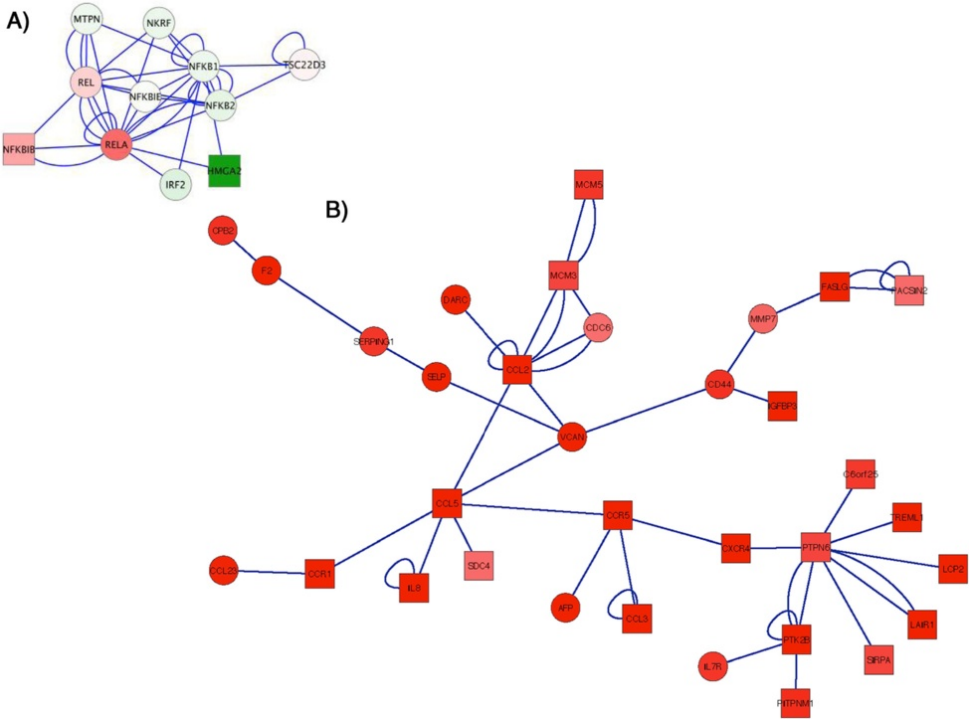
Genes changed by Meth in connection with CCR5 in rhesus monkey’s brain-derived immune cells. **a** Network containing transcription factors that have binding sites in the CCR5 promoter. **b** CCR5 centered network filtered for genes with expression above 1.5-fold. Circles indicate *p* > 0.05 and squares indicate *p* < 0.05

Thus, given the relevance for HIV CNS pathology and target availability, we focused on the pathways in connection with CCR5 upregulation by Meth. We searched for differences in gene expression of transcription factors that have binding sites within the CCR5 promoter in microglial from Meth and Control animals. We detected a cluster showing a 2.8-fold increase (*p* = 0.06) in p65 (RelA), a the NF-kappaB/Rel family member, that is a potent activator of the CCR5 promoters Pu and Pd [43] (Fig. 4a). This was accompanied by an up-regulation of NFKBIB (1.83-fold, *p* = 0.035), a NFKB deactivator that traps NF-kappa-B in the cytoplasm [44, 45] (Fig. 4a).

In order to identify genes in the data set connected to CCR5, that were also increased by Meth, we ran a node search that was centered on CCR5, contained first/second neighboring genes, that was filtered to expression levels increased above a 1.5-fold difference in Meth compared to control microglia (Fig. 4b). Using this approach we identified a large node showing the increase of the CCR5 ligand, CCL5/RANTES (3.7-fold, *p* = 0.0008) (Fig. 4b, Table 6) and other genes that exhibit pro-inflammatory properties (Table 6). This network, including second neighbors, was predominantly annotated to chemotaxis, calcium homeostasis, and inflammatory response (*p* < 0.0001, Benjamini < 0.0001). Other CCR5-related genes that can be important in pathogenesis included IL16, which can also bind to and attract CCR5-expressing cells [46], and Stat6, a marker of M2 phenotype in microglia [47].

**Table 6:** Genes significantly changed in connection with the upregulation of CCR5 in Meth compared to control microglia

CanonicalName	Meth/Ctr FC	Meth/Ctr pvalue	Name
CCL5	3.664732509	0.0008	Chemokine (C-C motif) ligand 5
RALGPS2	2.894990056	0.0206	Ral GEF with PH domain and SH3 binding motif 2
CCL3	2.632055343	0.0160	Chemokine (C-C motif) ligand 3
IL8	2.330578386	0.0152	Interleukin 8
KIF23	2.32350165	0.0090	Kinesin family member 23
PRPF19	2.277978004	0.0486	PRP19/PSO4 pre-mRNA processing factor 19 homolog (S. cerevisiae)
**CCR5**	**2.244743162**	**0.0149**	Chemokine (C-C motif) receptor 5
IL16	2.22856945	0.0025	Interleukin 16 (lymphocyte chemoattractant factor)
TGFB1	2.094232364	0.0181	Transforming growth factor, beta 1
PTK2B	2.075586714	0.0239	PTK2B protein tyrosine kinase 2 beta
CXCR4	2.290261762	0.0151	Chemokine (C-X-C motif) receptor 4
CCL2	1.976229643	0.0052	Chemokine (C-C motif) ligand 2
CCR1	1.944997932	0.0463	Chemokine (C-C motif) receptor 1
FGFR2	1.803077177	0.0075	Fibroblast growth factor receptor-2
PIN1	1.76086754	0.0429	Peptidylprolyl cis/trans isomerase, NIMA-interacting 1
VDAC1	1.745554071	0.0238	Voltage-dependent anion channel 1; similar to voltage-dependent anion channel 1
SIRPA	1.655090623	0.0180	Signal-regulatory protein alpha
PTPN6	1.651490977	0.0182	Protein tyrosine phosphatase, non-receptor type 6
AP2A1	1.648603535	0.0347	Adaptor-related protein complex 2, alpha 1 subunit
CRTC2	1.612923593	0.0216	CREB regulated transcription coactivator 2
ITGB5	1.580576538	0.0274	Integrin, beta 5
AR	1.575448506	0.0400	Androgen receptor
TNFAIP3	1.563086606	0.0044	Tumor necrosis factor, alpha-induced protein 3
BTRC	1.552901893	0.0263	Beta-transducin repeat containing
C5orf25	1.542952379	0.0097	Chromosome 5 open reading frame 25
NAGPA	1.524456014	0.0027	N-acetylglucosamine-1-phosphodiester alpha-N-acetylglucosaminidase
SDC4	1.523650476	0.0487	Syndecan 4
STAT6	1.482833897	0.0044	Signal transducer and activator of transcription 6

A network that includes significantly upregulated first and second neighbors of CCR5, which appears in bold, was generated. Values represent fold change in Meth versus Control microglia and *p* value

The upregulation of CCR5 and its neighboring genes by Meth is in agreement with our previous findings [19, 48], and provides associated molecules that may support viral targets in Meth abuse. Therefore, we have selected CCR5 and its co-regulators, as well as other markers annotated to inflammatory cell migration, to validate the implementation of our strategy, towards identifying relevant pathways from large sets of genes in SIV and Meth co-morbidities in vivo.

**Fig. 5:**
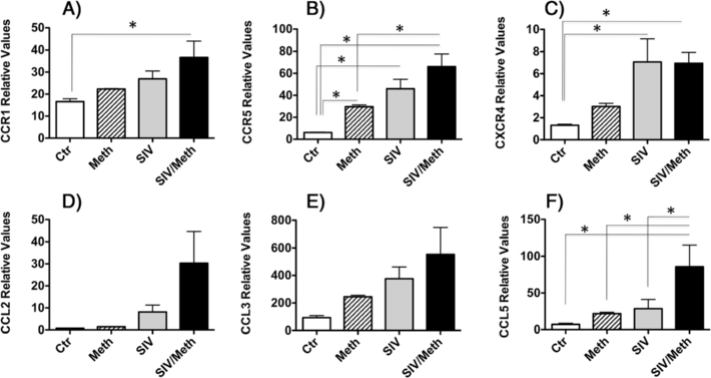
Effect of Meth and SIV on the expression levels of CCR5 and its associated chemokine and chemokine receptor genes in microglia. The expression of **a** CCR1, **b** CCR5, **c** CXCR4, **d** CCL2, **e** CCL3 and **f** CCL5 were measured using qRT-PCR. Expression levels were normalized to the expression of GAPDH. Values represent the average ± SEM. *N* = 4 animals/group. *p ≤ 0.05 in comparisons shown in lines (One-way ANOVA, followed by Bonferroni’s post-hoc test)

The expression of genes with a role in inflammatory cell migration in the CCR5 network was validated via qRT-PCR using samples from brain-derived microglia (Fig. 5). The genes tested were CCR1, CCR5, CXCR4, CCL2 (MCP-1), CCL3 (MIP-1a), and CCL5 (RANTES). Of these molecules, only CCR5 was confirmed by qRT-PCR to be significantly increased by Meth in comparison to control brain-derived microglia cells (Fig. 5b). However, the expression of the other genes examined was substantially enhanced by Meth, both in controls and in SIV-infected brain isolates. CCL5 (RANTES), in particular, was significantly enhanced by Meth in SIV-infected cells (Fig. 5f). Overall, this suggests that Meth enhances microglial activation and the expression of pro-inflammatory factors, especially those associated with susceptibility to viral infection. The correlation between CCR5 and susceptibility to HIV infection in Meth abuse has been attributed to the elevation of dopamine levels [49].

**Fig. 6:**
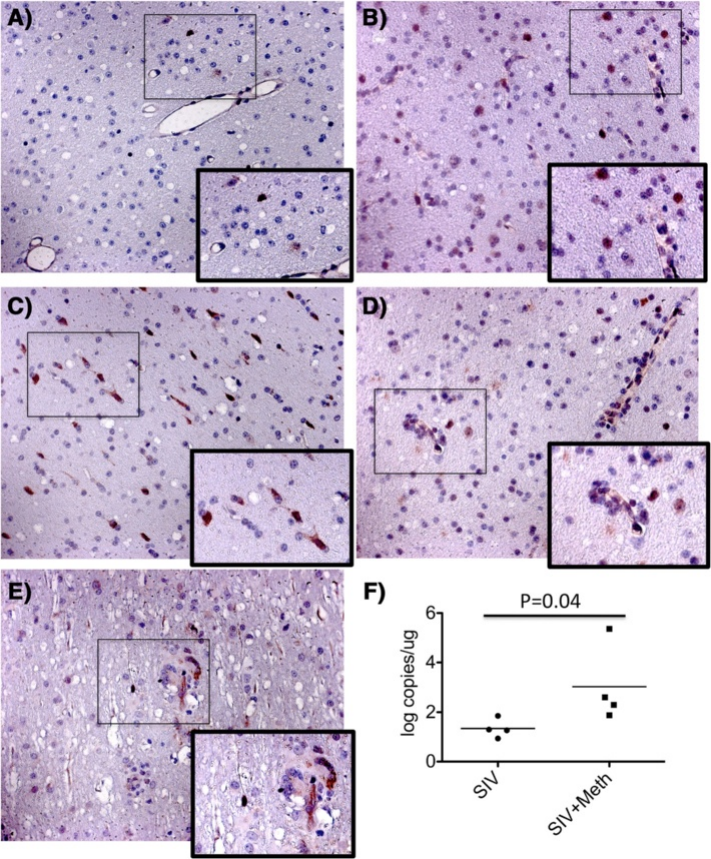
Detection of CCR5 and virus on brain tissue. CCR5 expression was analyzed using Immunohistochemistry on the layer III of frontal cortex from macaques that were **a** Uninfected and untreated controls, **b** Meth-only treated, **c** SIV-only infected, and **d** SIV-infected and Meth-treated. **e** SIV-infected and Meth-treated. Pictures were at 16× magnification. Sites of interest (rectangles) were further magnified. **f** Brain viral load (Student’s *t* test)

An in depth analysis of the pathway leading to an increase in CCR5-positive virus target cells in the brain [26] revealed additional, connected, cell migration pathways. The quest for neighboring CCR5 genes that were upregulated by Meth alone, led to a network of components associated to inflammatory cell migration and development of pro-inflammatory conditions. Of these, chemokines and chemokine receptors were validated, confirming the ability of Meth to favor the development of inflammatory pathologies and increasing the availability of virus target cells. The increase of CCR5 and its distribution in brain frontal lobe tissue were examined via immunohistochemistry. In controls we found that CCR5 is expressed by few cells exhibiting myelomonocytic morphology (Fig. 6a), while Meth-treatment increased the intensity and number of CCR5-positive cells perivascularly and at the parenchyma (Fig. 6b). As expected, SIV infection also increased the number of cells that are highly positive to CCR5 (Fig. 6c). In addition, Meth and SIV had an additive effect regarding the expression of CCR5-positive cells (Fig. 6d), including multinucleated giant cells (Fig. 6e), suggesting that moderate encephalitis occurred in correlation with increased brain viral load in those animals (Fig. 6f). The increase in CCR5 expression was confirmed by image analysis, using Image J. The measurements of CCR5-positive staining intensity, converted into percentage of the total area, revealed a significant increase in CCR5 tissue expression induced both by Meth alone (2.48 % ± 1.16) and by SIV alone (3.15 % ± 0.87), when compared to controls (0.91 % ± 0.35), with *p* values = 0.01 and 3.73E-05, respectively. SIV/Meth showed the increased effect of interaction (5.72 % ± 2.11), and CCR5 staining was significantly higher in that group, when compared to Meth alone (*p* = 0.0047) and to SIV alone (*p* = 0.0025).

Interestingly, the increase of viral load in the brain of Meth-treated SIV-infected animals compared to SIV did not correlate with viral levels in the plasma, or CSF (not shown), confirming a previous report [19]. This observation is in support of studies suggesting that viral load in brain parenchyma correlates with the development of overt neurological functions, when compared to plasma [50]. However, it has been suggested that viral antigen contributed by viral production within the brain, not absolute amount of viral load in the CSF, correlates more accurately with the development of neurological disease [50]. Therefore, the levels of virus in the brain parenchyma may precede the elevation of CSF viral load. Alternatively, because viral load was measured in cell-free CSF samples, differences in CSF viral load between SIV and SIV/Meth could be due to changes in the number of infected cells rather than free circulating RNA. Another explanation for a higher viral load in the brain, but not in the CSF, of Meth-treated animals could be potential disruptions in bi-directional flow and diffusion to intra-ventricular spaces.

Remarkably, the high viral load in the brain parenchyma associated to the finding that Meth abuse increases CCR5 expression has implications to the understanding of the epidemiology of HIV spread. Meth abusers, are not only more often at risk to become exposed to HIV, but also are immediately more susceptible to acquire infection and drive the virus towards the CNS. The ability of Meth to do this arises from its enhancement of pathways that lead to CCR5 upregulation in macrophages and glial cells. This finding may explain the high incidence of inflammatory pathologies in the brain and elsewhere among Meth abusers [51–53].

### D. The combination of Meth and SIV increases the expression of genes associated to encephalitis and disease progression

We were interested in identifying genes whose SIV-induced expression was potentiated by Meth, which could be predictive of an encephalitic outcome and could have a translational potential. In order to find genes with a severity predictive value, we compared the gene expression in brain cell isolates from Meth, SIV, and SIV/Meth microglia with previously characterized samples from SIV infected animals that had a severe encephalitis associated with fast disease progression (SIVE) [54]. The SIVE group differs from the SIV group in that it presents severe inflammatory pathology with strong diffuse and large focal inflammatory aggregates, which are not restricted to the perivascular domain, as well as multinucleated giant cells. The encephalitis in the SIVE group of animals developed spontaneously, as opposed to other models in which encephalitis is induced by CD8 depletion [55]. With such a comparison, we aimed at finding genes with expression levels potentiated by Meth and SIV to levels that were similar to what was observed in severe SIV inflammatory pathology.

**Fig. 7:**
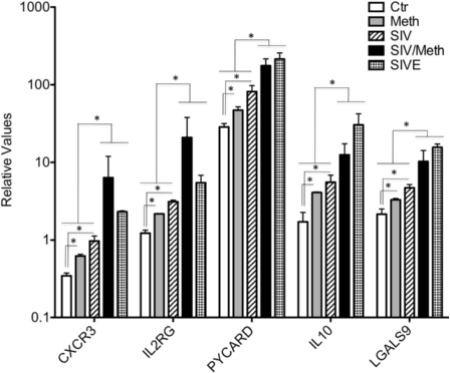
Gene expression of inflammatory molecules upregulated by both Meth and SIV co-morbidities that are also elevated in SIVE. These genes were selected based on the statistical significance observed in all groups compared to controls, between Meth or SIV individually and Meth/SIV as co-morbidities, but that were not different between SIV/Meth and SIVE, in gene array. *p ≤ 0.05 in comparisons shown in lines (One-way ANOVA, followed by Bonferroni’s post-hoc test)

The SIV-infected macaques that were treated with Meth developed mild to moderate encephalitis, with some perivascular infiltrate, eventual multinucleated giant cells, and microgliosis (Fig. 5), but did not exhibit the severe macrophage migration and parenchymal inflammatory foci typical of SIVE. They also presented an increase in five genes highly elevated in severe SIV cases of spontaneous encephalitis (Fig. 6). These genes were CXCR3 (IP10), IL2RG (IL2R common gamma chain), PYCARD (Apoptosis-associated speck-like protein containing a CARD), IL10, and LGALS9 (Galectin 9) (Fig. 7). This suggests that Meth and SIV, as co-morbidities, can increase levels of molecules associated with severity of CNS inflammation. Further proposing that Meth can induce changes in the CNS immune environment that increase predisposition to a severe inflammatory outcome. Of the genes that were commonly upregulated in SIVE and SIV/Meth, we have validated and confirmed the enhanced expression of CXCR3 (IP10 receptor), IL2RG (IL2 receptor common gamma chain), PYCARD, IL10 and LGALS9 (Galectin-9). These genes were significantly increased in SIV/Meth animals, compared to SIV only controls, in a similar fashion to the SIVE group. Individually, the identified genes have been described in correlation with brain inflammation. For instance, the transcriptional increase of IP10 and CXCR3 is common to several CNS inflammatory pathologies, including HIV [56, 57]. In addition, blocking CXCR3 reduces HIV-1 replication in vitro [57]. IL2RG is an important signaling component of many interleukin receptors, including IL15, which we have shown to be upregulated in the brain of SIV-infected macaques in correlation with homeostatic proliferation and persistence of pathogenic CD8 T cells [58]. Interestingly, over-expression of IL2RG has been linked to schizophrenia [59], which is one of the potential outcomes of mental dysfunction in association with HIV [60–62]. PYCARD is one of the components of the inflammasome, which is potentially activated by TLR-mediated viral recognition signaling pathways that enhance the inflammatory environment in the CNS [63–65]. Although IL10 is described as an immune-modulator, it has been shown to be a marker of HIV brain disease [66]. Finally, LGAL9 is a glycoprotein that is expressed in the brain by several innate immune cells [67, 68], and that can potentiate the infection in CD4 T cells that express a protein disulfide isomerase (PDI) ligand [69]. On the other hand, LGAL9 binding to another T cell ligand, T-cell immunoglobulin mucin domain 3 (Tim-3), can make cells more resistant to HIV infection by down-regulating co-receptors of viral entry [70]. However, in other models, the expression of LGAL9 is associated with apoptosis and a decreased capacity of T cells to perform immunity functions, in a Tim-3 dependent way [67, 68]. The role of this molecule in the brain, in the context of HIV is not clear. Nevertheless, the increase of its levels in microglia from both SIVE and SIV/Meth samples suggests that this molecule can be a marker of dysfunctional inflammatory pathways. Whether these genes are early markers of inflammatory severity remains to be examined.

Microglial cells are critical regulators of brain health and show pro-inflammatory phenotypes that may be detrimental to neuronal health. Activation of microglia and inflammation are associated to several neuropathologies, affecting neuronal behavior and survival while responding to local and systemic insults [71]. Isolation of microglia from the brain of SIV/Meth macaques allowed a clean evaluation of the changes in microglia that are associated with the aggravation of SIV-induced CNS disease caused by Meth exposure, which may reveal parallels with the analogous human condition. In fact, persistently activated microglia have been described in Meth users, even during periods of abstinence [18]. Microglial activation is in strong association with poor executive functioning [72] and the combination of Meth and HIV results in a worsening of cognitive functions [73]. The faster decay of CNS functions may partially result from poor adherence to anti-retroviral treatments [74]. However, it may also arise from the effects of the drug on the innate immune environment in the CNS, which may contribute to an elevated viral load, as observed by us in the SIV macaque model [19].

Our characterization of Meth induced transcriptional changes in microglia isolated from SIV-infected samples confirms the role of Meth abuse in promoting a pro-inflammatory environment in the brain. Meth treated samples featured an elevation of several molecules that facilitate the accumulation of inflammatory cells and phenotypic activation. For instance, genes in pathways of apoptosis, cell migration, and activation of T cells have been identified.

We found that hallmarks that distinguish relevant pathways of inflammation triggered by chronic Meth use were highlighted by an approach of isolation of immune cells from the brain. In addition, we confirmed the capacity of Meth to enhance CCR5-expressing targets cells for SIV in the brain. This finding potentially explains why increased viral load and neurotoxicity is found in SIV/Meth macaques and humans infected with HIV that consume Meth [26]. Collectively, the results suggest that changes caused by Meth in microglial cells play a significant role in the outcome of CNS disorders in the context of HIV infection.

## Conclusions

- Signals from particular groups of cells may be diluted in genomic analysis performed in whole brain tissue. The isolation of microglial cells to study changes in the gene expression profile that are caused by Meth, in the context of neuroAIDS, allowed a better analytical resolution to understand the effects of the drug on non-neuronal innate immune cell populations, and a focus on immunological parameters affecting the CNS environment, and in particular, interfering with susceptibility to infection and with CNS disease outcome towards SIV or HIV infection.
- Meth is one of the largest health problems associated to HIV exposure, and as a co-morbidity, increases CNS viral load in infected individuals. The examination of the effects of chronic Meth exposure in rhesus macaques showed that the drug altered the gene expression profile of CNS microglia cells and of brain-infiltrating immune cells, by promoting apoptotic and pro-inflammatory pathways, especially characterized by the enhancement of genes associated to chemokines and chemokine receptors. Within this pathway, the expression of CCR5 and its associated genes was largely relevant, due to the implications of this molecule in the susceptibility to SIV, which, like HIV, utilizes CCR5 as a co-receptor for entry into host’s cells in the brain. Therefore, the upregulation of CCR5 in Meth-exposed individuals increases the availability of virus targets, which may contribute to the higher viral load observed in the brain of SIV-infected, Meth-exposed animals, and of HIV-infected individuals.
- Meth treatment triggered the high expression of inflammatory markers that are also associated to rapid disease progression and cognitive decay, among them CXCR3, IL2RG, PYCARD, IL10 and LGALS9. This suggests that the drug induces potential pre-requirements associated to progression.

## Methods

### Monkeys and SIV infection

SIV-, simian retrovirus type D-, and herpes B virus-free rhesus macaques of Chinese origin purchased from Valley Biosystems (West Sacramento, CA) were infected with a cell-free SIV stock derived from SIVmac251 [75, 76]. All experiments had the approval from the Scripps Research Institute Animal Care and Use Committee and followed National Institutes of Health guidelines. Animals kept in containment were anesthetized with 10 to 15 mg/kg of ketamine intramuscularly before procedures. At necropsy, performed after terminal anesthesia, animals were intracardially perfused with sterile PBS containing 1 U/ml heparin. Brain tissue samples were taken for cell isolation, virus quantification, and formalin fixation for histology.

### Viral quantification

Brain SIV RNA was calculated by using a quantitative branched DNA signal amplification assay, performed by Siemens Clinical Laboratory (Emeryville, CA).

### Meth treatment

Meth was administered by intramuscular injection as previously described [77]. Briefly, Meth treatment was initiated at 19 weeks p.i. in animals with a stable plasma viral load. Escalating protocol was designed to mimic Meth abuse conditions, by administering the drug 5 days a week, twice a day, and further increasing the dose to reach a final dose of 2.5 mg/kg twice daily, after a 5-week ramp-up, for a total of 25 mg/kg/week [55, 77]. This level was then maintained for an additional 18 weeks. Three animals received PBS injections on the same schedule. All animals were sacrificed at 42 weeks p.i.

### Microglial cell isolation

The brain tissue was removed at necropsy after intravascular perfusion. For isolation of cells from the brain, the brain was carefully freed of meninges. Brain immune cells were isolated from all areas of the brain, by enzymatic digestion of minced tissue, followed by Percoll (Sigma-Aldrich) gradient, as described previously [27]. The isolated cells were quantified in a Z2 Coulter Counter (Beckman Coulter, Brea, CA), and further characterized by flow cytometry, using anti-CD11b (clone M1/70, BD Pharmingen, San Diego, CA), anti-CD45LCA (clone 2B11, BD Biosciences, San Diego, CA), anti-CD14-PE (clone M5E2, BD Pharmingen, San Diego, CA), anti-CD16-FITC (clone 3G8, BD Pharmingen), anti-monkey CD3-biotin (clone FN-18, Invitrogen Biosource, Carlsbad, CA) followed by streptavidin-PerCP or -APC (BD Pharmingen), anti-human CD8-PE, −FITC, or -PeCy5 (clone DK25, Dako, Carpinteria, CA), anti-CCR5, CCR2, CX3CR1, CD80 (BD Biosciences, San Jose, CA), and CD44v6 (clone 2 F10, Zymed, San Francisco, CA).or isotype controls (BD Pharmingen). Stained cells were acquired by a FACSCalibur (BD Biosciences, San Jose, CA) flow cytometer, and analyzed in FlowJo 6.2.1 software (Tree Star Inc., Ashland, OR), as previously described [27].

### Samples, labeling and gene array

Gene analysis was performed on cryopreserved microglial cells, by custom Miltenyi Biotec Array Services. All samples were individually performed in duplicate. RNA was isolated from all macaques using standard RNA extraction protocols (NucleoSpin RNA II, Macherey-Nagel). Quality of the samples was checked via Agilent 2100 Bioanalyzer platform (Agilent technologies). A RNA integrity number (RIN) was calculated by a proprietary algorithm that takes several QC parameters into account, such as 28S/18S RNA peak area ratios and unexpected peaks in the 5S RNA region, and RIN number of 10 indicates high quality, and 1 low quality. A RIN >6 is of sufficient quality for gene expression profiling experiments [78, 79]. All samples, except for 1 (animal 492 – Normal control) showed values above 6. That animal was excluded from the analysis, leaving the healthy control group with an *n* = 4. For the linear T7-based amplification step, 100 ng of each total RNA sample was used. To produce Cy3-labeled cRNA, the RNA samples were amplified and labeled using the Agilent Low Input Quick Amp Labeling kit (Agilent technologies) following the manufacturer’s protocol. Yields of cRNA and the dye incorporation rate were measured with the ND-1000 spectrophotometer (Nanodrop Technologies). The hybridization procedure was performed according to Agilent 60-mer 0ligo microarray processing protocol using the Agilent gene expression hybridization kit (Agilent Technologies). Briefly, 1.65ug Cy3-labeled fragmented cRNA in hybridization buffer was hybridized overnight (17 h, 65oC) to Agilent Whole Rhesus monkey Genome oligo microarrays 4×44K (one-color) using Agilent’s recommended hybridization chamber and oven. Finally, the microarrays were washed once with the Agilent Gene expression wash buffer 1 for 1 min at room temperature, followed by a second wash with pre-heated Agilent Gene expression wash buffer 2 (37oC) for 1 min. The last washing step was performed with acetonitrile. Fluorescent signals of the hybridized microarrays were detected using Agilent’s microarray Scanner System (Agilent Technologies). The Agilent Feature extraction software (FES) was used to read out and process the microarray image files, determining feature intensities (including background subtraction), rejecting outliers and calculating statistical confidences. For determination of differential gene expression FES derived output data files were further analyzed using Rosetta Resolver gene expression data analysis system (Rosetta Biosoftware), for comparing two single intensity profiles in a ratio experiment.

### Systems analysis

Pathway assignments and functional annotations were analyzed using DAVID Bioinformatics Database [20]. To complete the bioinformatics analysis, two knowledge base resources were queried: the Ingenuity Knowledge Base [21] and an interaction repository, which is based on cpath [22–24] and includes interactions that have been curated by GeneGo (http://portal.genego.com), the Kyoto Encyclopedia of Genes and Genomes (KEGG - http://www.genome.jp/kegg/), and Ingenuity. Benjamini False Discovery Rate (FDR) adjusted values <0.01 and *p* values < 0.05 (provided by DAVID) were utilized as conservative filters for identification of true values. Cluster analysis and networks were obtained and visualized using Cytoscape [25].

### Quantitative RT-PCR

First Strand kit (Qiagen) was used for cDNA synthesis. Primers were designed based on available sequences for Macaca mulatta in Gene Database for detection of relative levels using SyBrGreen/ROX in an ABI HT7900 machine. Data was analyzed with Sequence Detection System software and expressed in relative values following the normalization with GAPDH expression.

### Immunohistochemistry

Following perfusion, the brain tissue was fixed in 10 % buffered formalin for 48 h, followed by 70 % ethanol. Tissues were embedded in paraffin, cut into 5 μm sections, and mounted on glass slides. Rehydrated sections were blocked to endogenous peroxidase activity by treating slides with 3 % hydrogen peroxide in absolute methanol. Following that, the slides were placed in a solution of 0.01 M Citrate, pH 6.39, in a humidified heated chamber, for antigen exposure. Sections were blocked with 5 g/l Casein (Sigma Aldrich) in PBS, containing 0.5 g/l Thimerosal (Sigma Aldrich) and incubated with Iba-1 (Wako Lab Chemicals, Richmond, VA) or the anti-human CCR5 (NBP2-31374, Novus Biologicals, Littleton, CO), each one diluted in Casein buffer. Biotinylated goat anti-rabbit IgG antibodies (Vector Labs, Burlingame, CA) were used at a 1/300 dilution. Visualization was achieved using biotin/avidin-peroxidase (Vector Labs) and Nova Red (Vector Labs). Counterstaining was made with Gill’s hematoxylin. Images were captured using an Axiovert 200 inverted microscope (Carl Zeiss) with Axio Vision software (version 4.8.1; Carl Zeiss). Image analysis was performed in Image J 6.4 (NIH, USA). For that, tiff image files were opened and manually thresholded to identify stained cells. A binary mask was obtained from the negative thresholded image and measurement values were calculated as percentage of the total area. This was performed in a minimum of 5 fields per section, and two sections per animal.

### Statistics

Group comparisons were performed using the tests described in the text and figure legends. The difference between the means was considered significant at *P* < 0.05. Tests were performed using Excel (Microsoft Corporation, Redmond, WA) and Prism software (GraphPad Software Inc., San Diego, CA) for Macintosh.

### Ethics approval and consent to participate

All experiments had the approval from the Scripps Research Institute Animal Care and Use Committee and followed National Institutes of Health guidelines.

### Availability of data and material

The data supporting our findings can be found in Additional files 1, 2 and 3: Table S1, S2 and S3.

## Additional files

Additional file 1: Complete list of genes upregulated in microglia isolated from Meth-treated animals compared to controls. Genes in this list are increased above 1.5-fold in Meth versus control, with a *p* value below 0.05. (XLSX 167 kb) Additional file 2: Complete list of genes upregulated in microglia isolated from SIV-infected animals compared to controls. Genes in this list are increased above 1.5-fold in SIV versus control, with a *p* value below 0.05. (XLSX 306 kb) Additional file 3: Complete list of genes upregulated in microglia isolated from animals that are SIV-infected and Meth-treated compared to SIV-infected animals. Genes in this list are increased above 1.5-fold in MethSIV versus SIV, with a *p* value below 0.05. (XLSX 69 kb)

## Abbreviations

AIF1: Allograft inflammatory factor 1
ANOVA: analysis of variance
CCR1: C-C chemokine receptor type 1
CCR2: C-C chemokine receptor type 2
CCR5: C-C chemokine receptor type 5
CNS: central nervous system
CSF: Cerebral Spinal Fluid
CXCR4: C-X-C chemokine receptor type 4
FasLG: Fas Ligand
FES: feature extraction software
HIV: human immunodeficiency virus
IFNg: interferon gamma
IL10: interleukin 10
IL2RG: interleukin 2 receptor common gamma chain
IP10: IFNg-inducible protein 10
KEGG: Kyoto encyclopedia of genes and genome
LCP2: lymphocyte cytosolic protein 2
LFA2: lymphocyte function associated antigen 2
LGALS9: Galectin 9
MCL: Markov Clustering Algorithm
Meth: methamphetamine
NFKB: nuclear factor kappa B
NFKBIB: NF-kappa-B inhibitor beta
NOD1: nucleotide-binding oligomerization domain-containing protein 1
PYCARD: Pyrin and CARD domain-containing protein
RANTES: regulated on activation, normal T cell expressed and secreted
RIN: RNA integrity number
SH3BP2: SH3 domain binding protein 2
SIV: Simian immunodeficiency virus
SIVE: SIV encephalitis
SLA: Src-linked adaptor
SOCS1: suppressor of cytokine signaling 1
TCR: T cell receptor
TNFa: tumor necrosis factor alpha
TUBA1B: tubulin alpha 1B
